# Supplementary material for: Can serum interleukin 34 levels be used as an indicator for the prediction and prognosis of COVID-19?
Source: PLoS One. 2024 Apr 16;19(4):e0302002. doi: 10.1371/journal.pone.0302002 (PMC11020891; doi:10.1371/journal.pone.0302002)

SAMPLING

The sample size of this study was determined by power analysis. According to the calculation made using the G*power 3.1 program; the sample size was determined to be at least 80 (40 in each group) with an effect size of 0.75, margin of error of 0.05, confidence level of 0.95, and representative power of the universe of 0.95 (Faul, Erdfelder, Burchner ve Lang, 2009).

Faul, F., Erdfelder, E., Lang, A.-G., (2009). G*Power 3.1: Test For Correlation and Regression Analyses Flexible Statistical Power analysis Behavior Research Methods, 41, 1149-1160.

APPENDIX- G.Power Program Output

t tests – Means: Difference between two independent means (two groups)

**Analysis:**	A priori: Compute required sample size		
**Input:**	Tail(s)	=	One
	Effect size d	=	0.75
	α err prob	=	0.05
	Power (1−β err prob)	=	0.95
	Allocation ratio N2/N1	=	1
**Output:**	Noncentrality parameter δ	=	3.3541020
	Critical t	=	1.6646246
	Df	=	78
	Sample size group 1	=	40
	Sample size group 2	=	40
	Total sample size	=	80
	Actual power	=	0.9535156

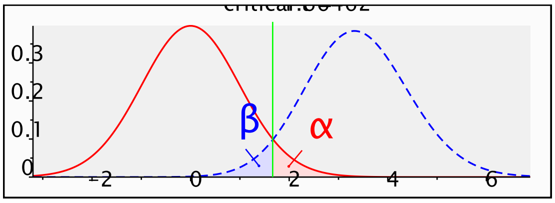

Supplement: S1 File — (PDF) [file pone.0302002.s002.pdf]
