# Supplementary material for: Can serum interleukin 34 levels be used as an indicator for the prediction and prognosis of COVID-19?
Source: PLoS One. 2024 Apr 16;19(4):e0302002. doi: 10.1371/journal.pone.0302002 (PMC11020891; doi:10.1371/journal.pone.0302002)
Supplement: Attachment — Submitted filename: Response to reviewers plos one 2.docx [file pone.0302002.s004.docx]

Dear Editor,

We thank you for your kind reply to our paper (PONE-D-23-19398) entitled " CAN SERUM INTERLEUKIN 34 LEVELS BE USED AS AN INDICATOR FOR THE PREDICTION AND PROGNOSIS OF COVID-19?’’. We have evaluated your precious comments on our manuscript and have revised our article in light of your suggestions. Below you can find our answer. We will be very pleased if you re-evaluate our work. 21.03.2024

Kind regards.

Doğu Karahan

Corresponding author

[dogu.karahan@ozal.edu.tr](mailto:dogu.karahan@ozal.edu.tr)

Journal Requirements:

Please review your reference list to ensure that it is complete and correct. If you have cited papers that have been retracted, please include the rationale for doing so in the manuscript text, or remove these references and replace them with relevant current references. Any changes to the reference list should be mentioned in the rebuttal letter that accompanies your revised manuscript. If you need to cite a retracted article, indicate the article’s retracted status in the References list and also include a citation and full reference for the retraction notice.

Our answer: References have been checked. The doi number of reference number 1 has been added. No additional abnormalities were observed in the references.

Reviewer 1:

Dear reviewer, thank you for your evaluation and suggestions. We have made adjustments in line with your suggestions. We will be very pleased if you re-evaluate our work.

Kind regards.

1. How come the ICU patients included in the study did not receive dexamethasone?

Our answer: Dexamethasone treatment was administered to intensive care COVID-19 patients. However, IL-34 levels were measured before the first dexamethasone administration. The cases included in the study from ICU were the cases who were admitted to the intensive care unit within the first 24 hours after our COVID-19 clinical or emergency service admission. After blood samples were taken, dexamethasone treatments were administered to the patients. When we reviewed the article again, we realized that we could not explain this part clearly, and we added it more clearly to the material and method section as you suggested.

2. Were the autoimmune or cancer history evaluated for the included patients to see if either is contributing to the elevated IL-34? While this question was partly clarified, did any of the patients get serologic testing for antibodies? Or were assumed to be negative based on clinical history. Especially with COVID having a lot of respiratory and renal failure similar to a lot of pulmonary/renal syndromes associated with vasculitis, were they definitely ruled out?

Our answer: It was decided that the cases were not diagnosed as auto-immune or cancer, based on their medical history and clinical status. Since there was no clinical suspicion in the follow-up of the cases, serological testing or further examination and evaluation were not performed in these respects. You are right that there could be many overlaps with COVID-19, similar to vasculitic lung/kidney syndrome. No detailed evaluation has been made on this issue. If you wish, we can add this situation to the limitations of the study section.

3. I would recommend some of the explanations here be incorporated into the article for better clarity.

Our answer: When we reviewed the article again, we realized that we could not clearly explain the parts you mentioned above, and we added it more clearly to the material and method section as you suggested.
